# Tissue-resident memory Th17 cells maintain stable fungal commensalism in the oral mucosa

**DOI:** 10.1038/s41385-020-0327-1

**Published:** 2020-07-27

**Authors:** Florian R. Kirchner, Salomé LeibundGut-Landmann

**Affiliations:** 1 Section of Immunology, Vetsuisse Faculty, University of Zürich, Winterthurerstrasse 266a, CH-8057 Zürich, Switzerland; 2 Institute of Experimental Immunology, University of Zürich, Winterthurerstrasse 190, CH-8057 Zürich, Switzerland

## Abstract

Keeping a stable equilibrium between the host and commensal microbes to which we are constantly exposed, poses a major challenge for the immune system. The host mechanisms that regulate homeostasis of the microbiota to prevent infection and inflammatory disorders are not fully understood. Here, we provide evidence that CD4^+^ tissue-resident memory T (T_RM_) cells act as central players in this process. Using a murine model of *C. albicans* commensalism we show that IL-17 producing CD69^+^CD103^+^CD4^+^ memory T cells persist in the colonized tissue long-term and independently of circulatory supplies. Consistent with the requirement of Th17 cells for limiting fungal growth, IL-17-producing T_RM_ cells in the mucosa were sufficient to maintain prolonged colonization, while circulatory T cells were dispensable. Although T_RM_ cells were first proposed to protect from pathogens causing recurrent acute infections, our results support a central function of T_RM_ cells in the maintenance of commensalism.

## Introduction

All epithelial barrier tissues are colonized with complex microbial communities that modulate the balance between health and disease. Maintaining a stable equilibrium with the microbiota represents a major challenge for the immune system. The relevance of fungi as an integral part of the microbiota has recently gained attention.^1^ Active and continuous surveillance of commensal fungi by the immune system is critical for host homeostasis and to prevent disease.^2,3^ In turn, a breach in host defenses e.g. as a consequence of immunosuppression, increases the susceptibility to fungal infections. Overall, fungal infections are a leading cause of disease worldwide and the frequency of reported incidences is on the rise.^4^

Amongst the most abundant human commensal fungi are those of the genus *Candida* with the most well-known species *Candida albicans*, colonizing the mucosae of the oral,^5,6^ gastrointestinal^7,8^ or urogenital tract^9^ and skin.^10^ *Candida*-mediated diseases manifest most frequently as superficial infections that affect the mucosal surfaces, the skin and nails.^11^ More severe forms of disease occur if the fungus reaches the bloodstream and disseminates to visceral organs, where it can cause life-threatening infections that are difficult to diagnose and treat.^12^ Colonization of epithelial barriers with *Candida* spp. has also been associated with an increased risk for inflammatory pathologies under certain conditions. As such, inflammatory bowel disease, a common inflammatory disorder of the gastrointestinal tract, has been linked to fungal dysbiosis^13,14^ and single nucleotide polymorphisms in genes that are involved in fungal recognition and response have been identified as risk factors for disease.^15–17^ *Candida* colonization of the gastrointestinal tract has also been linked to allergic disorders at distant sites such as the airways.^18,19^

These examples illustrate the necessity that fungal commensalism is tightly controlled. Our understanding of the immunosurveillance mechanisms that maintain stable colonization and prevent disease in epithelial tissues remains incomplete. The key role of CD4^+^ T cells in protection from *C. albicans* overgrowth has become apparent through the increased frequency of severe and chronic forms of superficial *C. albicans* infections in individuals with inherited or acquired T cell deficiencies, such as severe combined immunodeficiency or acquired immune deficiency syndrome, respectively.^20,21^ Among CD4^+^ T cells, especially those secreting IL-17 are critical for control of *C. albicans*. Individuals with primary immunodeficiencies resulting in defective Th17 immunity, such as *STAT3* loss-of-function (LOF) mutations in autosomal-dominant hyper-IgE syndrome, *STAT1* gain-of-function mutations, *RORC* LOF mutations, or *CARD9* LOF mutations show a strongly increased risk of developing chronic mucocutaneous candidiasis (CMC).^22–30^ Genetic defects in the IL-17 pathway itself, such as mutations in the IL-17 receptor A (IL-17RA) or IL-17RC subunits, in the signaling component Act1 or in the cytokine IL-17F were also found to underlie CMC.^31–33^

The key role of IL-17-mediated antifungal immunity has been recapitulated in mouse models of oropharyngeal candidiasis (OPC) and epicutaneous candidiasis. Mice lacking IL-17A and IL-17F or components of the IL-17 receptor (*Il17ra*^*−/−*^ and *Il17rc*^*−/−*^ mice) display strongly increased fungal loads compared to control mice.^34–40^ The same also applies to mice that lack factors required for the development of IL-17 secreting cells (*Rorc*^*−/−*^, *Il23p19*^*−/−*^mice).^34,37,40^ In addition to T cells, innate lymphoid cells and γδ T cells have also been implicated in IL-17-dependent antifungal immunity in mice, in particular at the onset of infection,^34,40–42^ as most studies so far employed models of acute *C. albicans* infection.

The species of *C. albicans* displays a large genetic diversity,^43^ which translates into varying degrees of fitness and virulence of the fungus. Mouse models of *C. albicans* infection facilitated the revelation of functional differences between individual strains in a uniform and *C. albicans*-naive host background. We and others have shown that this intra-species diversity of *C. albicans* greatly affects the pathogenicity of the fungus and the outcome of its interaction with the host.^44–46^ Highly virulent strains of *C. albicans*, such as the commonly used lab strain SC5314, infect the mucosal tissue of experimentally infected mice only transiently and get rapidly cleared as a result of their capacity to cause damage to epithelial cells. Host cell damage triggers a strong inflammatory response that is characterized by massive cytokine production and influx of neutrophils and monocytes to the site of infection.^35,45,47^ In contrast, *C. albicans* strains with a low capacity to induce epithelial cell damage induce only a limited degree of inflammation in the tissue and instead persist for a prolonged period of time in the murine oral mucosa.^45^ The lack of a rapid inflammatory host response by these strains is not the consequence of overt immunosuppression by regulatory T cells or IL-10,^48^ but rather due to *C. albicans*-intrinsic differences in the virulence of these strains albeit the underlying genetic basis remains unclear to date.

Although the host response to high- and low-virulent strains of *C. albicans* displays major qualitative and kinetic differences at the onset of infection, activation of *C. albicans*-specific Th17 immunity is conserved across isolates.^45,48^ Importantly, IL-17 signaling is essential for regulating stable colonization and for preventing fungal overgrowth, irrespective of the strain of *C. albicans* analyzed.^45^

To fathom the mechanisms that ensure continuous immunosurveillance of *C. albicans* during commensalism, we made use of a new experimental model of *C. albicans* persistent colonization in mice using the low-virulent strain 101, which closely mimics the situation in humans. The phenotypic and functional analysis of the dynamics and qualities of the Th17 response in the colonized epithelial tissue revealed a key role of tissue-resident memory T (T_RM_) cells that are maintained independently of the circulating T cell pool and that are critical for preventing uncontrolled outgrowth of the commensal fungus in the oral mucosa.

## Results

### T cells are required for regulating fungal colonization in the oral mucosa

**Fig. 1:**
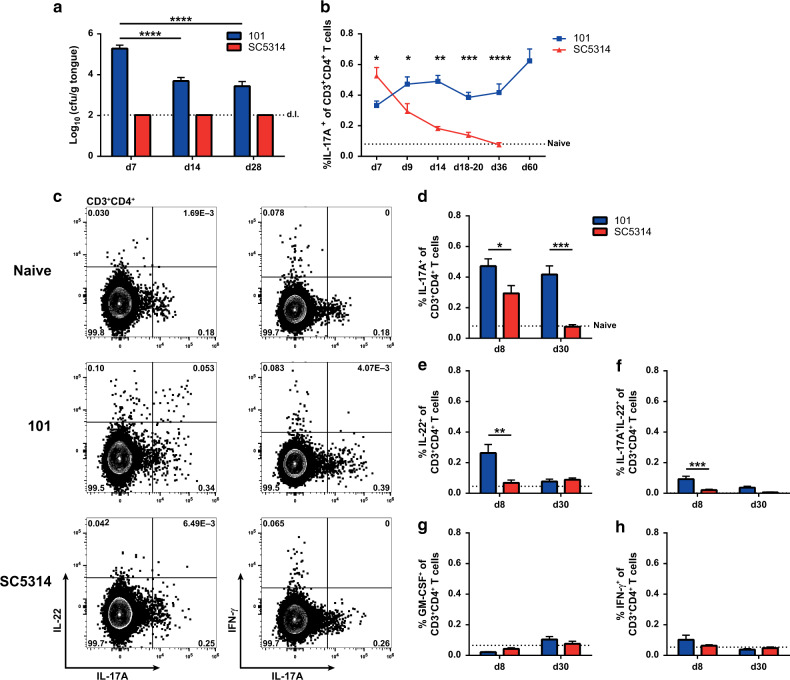
Th17 cell responses are sustained during stable colonization with *C. albicans*. WT mice were infected sublingually with *C. albicans* strain 101 (blue) or strain SC5314 (red) for the indicated periods of time. **a** The fungal burden in the tongue was determined by plating tissue homogenates on YPD agar. Data are the mean + SEM of 7–16 individual mice per group pooled from 2–4 independent experiments. The dotted line represents the detection limit (d.l.). **b–h** Cervical lymph node cells were re-stimulated with *C. albicans*-pulsed DC^1940^ cells for 5 h in the presence of Brefeldin A. IL-17A, IL-22, GM-CSF, and IFN-γ production was analyzed by intracellular cytokine staining and flow cytometry. Plots show the frequency of cytokine-producing CD3^+^CD4^+^ cells at the indicated time points (**b, d–h**) or representative FACS plots for IL-17, IL-22, and IFN-γ on day 8 post infection, respectively (**c**). In (**b)** data are the mean + SEM of 6–23 mice per group pooled from 2–7 independent experiments. In (**d–h)**, each bar represents the mean + SEM of 8–20 individual mice per group pooled from 2–5 independent experiments. The dotted line represents the mean value determined in naive mice. Statistics were calculated using two-way ANOVA *p < 0.05, **p < 0.01, ***p < 0.001, ****p < 0.0001. See also Supplementary Fig. S1.

The experimental model of murine OPC was used extensively to characterize the host response to *C. albicans* in mucosal barrier tissues. However, as in other infection models, the widely used highly virulent strain SC5314 is rapidly cleared from the oral cavity^34,37^ and this precludes monitoring the adaptive immune response against the fungus over time. The observation that the low-virulent *C. albicans* strain 101 persists for several weeks in the oral mucosa, in some cases up to 1 year,^45,48^ opens new opportunities for assessing the immunosurveillance mechanisms that maintain stable fungal colonization of the host tissue. Although fungal loads remained high in the tongue of strain 101-colonized mice during the entire observation period, we noticed a significant decline between day 7 and 14 post infection (Fig. 1a), which suggested an involvement of the adaptive immune system in the control of the colonization load. Indeed, *Rag1*^*−/−*^ and *Tcrbd*^*−/−*^ mice, which lack T cells, displayed a significantly higher fungal burden on day 7 and day 14 after infection with strain 101, compared to WT mice (Supplementary Fig. S1A–D). Of note, CD4^+^ T cells contributed to the maintenance of stable colonization with *C. albicans* in experimentally infected mice also beyond the first 2 weeks of infection. Moreover, adoptive transfer of *C. albicans*-induced polyclonal CD4^+^ T cells or *C. albicans*-specific TCR-transgenic Hector CD4^+^ T cells^49^ into *Rag1*^*−/−*^ mice prior to colonization with strain 101 was sufficient to rescue fungal control, with a significant proportion of the transferred cells producing IL-17 and IL-22 in response to *C. albicans* in either case (Supplementary Fig. S1E–H). Finally, we confirmed the relevance of IL-17A and IL-17F for retaining stable fungal loads during colonization over time (Supplementary Fig. S1I). Overall, the situation in *C. albicans* strain 101-colonized mice is reminiscent of the situation in humans, where a defect in the T cell compartment results in uncontrolled growth of the fungus and in disease.^20^

### Colonization with *C. albicans* strain 101 induces a sustained Th17 cell response

Consistent with the requirement of an intact IL-17 pathway for control of *C. albicans* colonization in humans, the fungus induces a prominent and selective Th17 response during experimental OPC.^45,49–51^ In response to the highly virulent strain SC5314, we found the population of *C. albicans*-specific Th17 cells to contract rapidly after fungal clearance. In contrast, in mice infected with the low-virulent strain 101, fungus-specific Th17 cells were maintained at high frequencies in the cervical lymph nodes at all time points analyzed (Fig. 1b–d).

We then characterized the cytokine profile of *C. albicans*-specific Th17 cells in the cervical lymph nodes of strain 101-colonized mice in more detail. On day 8 post infection, we observed a prominent population of IL-22-producing T cells, a fraction of which co-produced IL-22 and IL-17 (Fig. 1c, e, f), although IL-22 production was transient and low at later time points (Fig. 1e, f). In contrast, *C. albicans*-specific T cells expressed only very limited levels of GM-CSF and IFN-γ at any time point analyzed (Fig. 1g, h). Together, these results show that sustained colonization of the oral mucosa with *C. albicans* is accompanied with a selective Th17 response that is stably maintained.

### CD4^+^ T cell dynamics in the *C. albicans*-colonized tongue

**Fig. 2:**
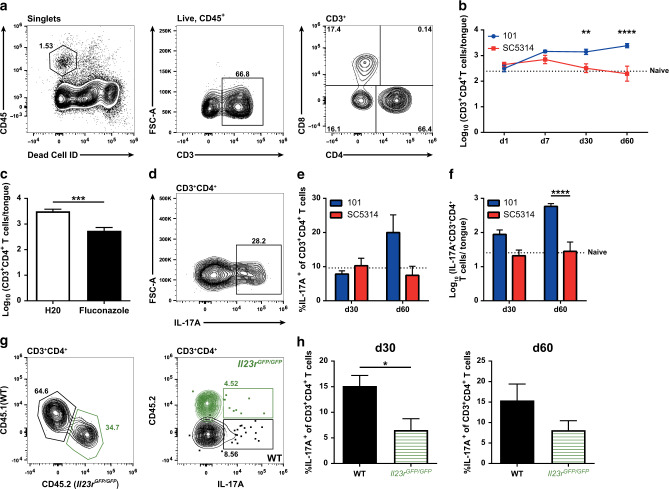
Th17 cells are maintained in the tongue during stable colonization with *C. albicans*. WT mice were infected sublingually with *C. albicans* strain 101 (blue) or strain SC5314 (red), respectively, for the indicated periods of time. **a** Representative FACS plots show the gating strategy for tongue CD3^+^CD4^+^T cells. The example shown is from a strain 101-infected mouse on day 60 post infection. **b** Quantification of tongue CD4^+^ T cells at the indicated time points post infection. Each symbol represents the mean + SEM of 7–13 individual mice per group pooled from 3–4 independent experiments. **c** Mice infected sublingually with strain 101 were or were not treated with fluconazole starting from day 8 post infection for 14 days. 60 days post infection, CD3^+^CD4^+^ T cells in the tongue were quantified by flow cytometry. Data are the mean + SEM from 7–13 individual mice per group pooled from 3 independent experiments. **d–f** Tongue cells that were isolated on day 30 or day 60 post infection and re-stimulated ex vivo with PMA and ionomycin for 4 h in the presence of Brefeldin A. L-17A production by CD3^+^CD4^+^ T cells was analyzed by intracellular cytokine staining and flow cytometry. A representative FACS plot is shown in (**d)**; quantification of the frequency (**e**) and total numbers (**f**) of IL-17^+^CD3^+^CD4^+^ T cells in the tongue. Each bar represents the mean + SEM of 7–10 individual mice per group pooled from 2–3 independent experiments. **g**, **h** CD45.1^+^CD45.2^+^ WT recipient mice were irradiated and reconstituted with 1:1 mixed bone marrow from CD45.1^+^ WT and CD45.2^+^ *Il23r*^*GFP/GFP*^ donor mice. After 8 weeks of reconstitution, chimeric mice were infected sublingually with *C. albicans* strain 101 for 30 and 60 days. IL-17A production by tongue CD3^+^CD4^+^ T cells was analyzed by intracellular cytokine staining and flow cytometry after re-stimulation ex vivo with PMA and ionomycin for 4 h in presence of Brefeldin A. Representative FACS plots of tongue CD4^+^ T cells in either compartment are shown in (**g**); quantification of the frequency of IL-17^+^ CD4^+^ T cells in either compartment are shown in (**h**). Each bar represents the mean + SEM of 6–7 individual mice pooled from two independent experiments. The dotted lines in **b**, **e**, and **f** represent the mean value determined in naive mice. Statistics were calculated using *t*-test (**c**, **h**) or two-way ANOVA (**b**, **e**–**f**), **p < 0.01, ***p < 0.001, ****p < 0.0001. See also Supplementary Fig. S2.

Next, we sought to characterize the local T cell response in the tongue of stably colonized mice. For this, we made use of a protocol that was previously established in our lab to isolate tongue T cells, which are rare in this intractable organ.^41,52^ Starting from 1 week post infection, a small but distinct population of CD4^+^ T cells was detected in the tongue of mice infected with *C. albicans* strain 101 or strain SC5314 (Fig. 2a, b). In line with the rapid clearance of strain SC5314 and the concomitant transient nature of the Th17 response in the cervical lymph nodes (Fig. 1), the CD4^+^ T cell population in the tongue also rapidly declined (Fig. 2b). In contrast, during persistent colonization of the oral mucosa with strain 101, high numbers of CD4^+^ T cells were stably maintained in the tongue throughout the analysis period (Fig. 2b), paralleling the sustained frequency of Th17 cells in the cervical lymph nodes (Fig. 1). To confirm that the difference in tongue T cell dynamics was not the consequence of a putative strain-specific difference that might influence the longevity of the T cell response, but rather resulted from the differences in persistence of the two strains in the mucosal tissue, we simulated a transient infection with strain 101 by treating colonized mice with the antifungal drug fluconazole from day 8 post infection to eliminate the fungus (Supplementary Fig. S2A). Fungal clearance was accompanied with a rapid decline in T cells in the tongue, indicating that the effect was indeed strain-independent (Fig. 2c).

We also analyzed CD8^+^ T cells in the tongue of *C. albicans*-colonized animals. In contrast to CD4^+^ T cells, this T cell subset was not enriched in strain 101-compared to strain SC5314-infected animals. Except at a very late time point, the frequency of CD8^+^ T cells was not increased in stably colonized mice in comparison to those that had undergone an acute infection (Supplementary Fig. S2B).

Ex vivo re-stimulation of tongue CD4^+^ T cells revealed a pronounced IL-17 production on day 30 and even more pronounced on day 60 post infection with strain 101 (Fig. 2d–f), while in case of strain SC5314-infected animals, no IL-17 production above baseline was detectable at these time points. As in the lymph nodes, tongue CD4^+^ T cells did not produce IFN-γ in the *C. albicans*-colonized tongue.

IL-23 is a key regulator of the generation of Th17 cells.^53^ We therefore examined the relevance of IL-23 signaling for the Th17 response in the oral mucosa during *C. albicans* colonization. Because IL-23 deficiency impairs fungal control and this would likely have an indirect effect on the Th17 response, we generated mixed bone marrow chimeras using IL-23 receptor-deficient and -sufficient bone marrow to reconstitute WT hosts. This strategy allows uncoupling of the Th17 response from fungal burden. As expected, Th17 cell frequencies within the IL-23R-deficient CD4^+^ T cell compartment were strongly reduced in comparison to their IL-23R-sufficient counterpart in chimeric mice colonized with *C. albicans* strain 101 (Fig. 2g, h). Collectively, these results indicate that stable colonization of the oral mucosa with *C. albicans* results in the accumulation and stable maintenance of Th17 cells in the tongue in an IL-23-dependent manner.

### CD4^+^ T cells in the tongue of *C. albicans*-colonized mice display a T_RM_ phenotype

**Fig. 3:**
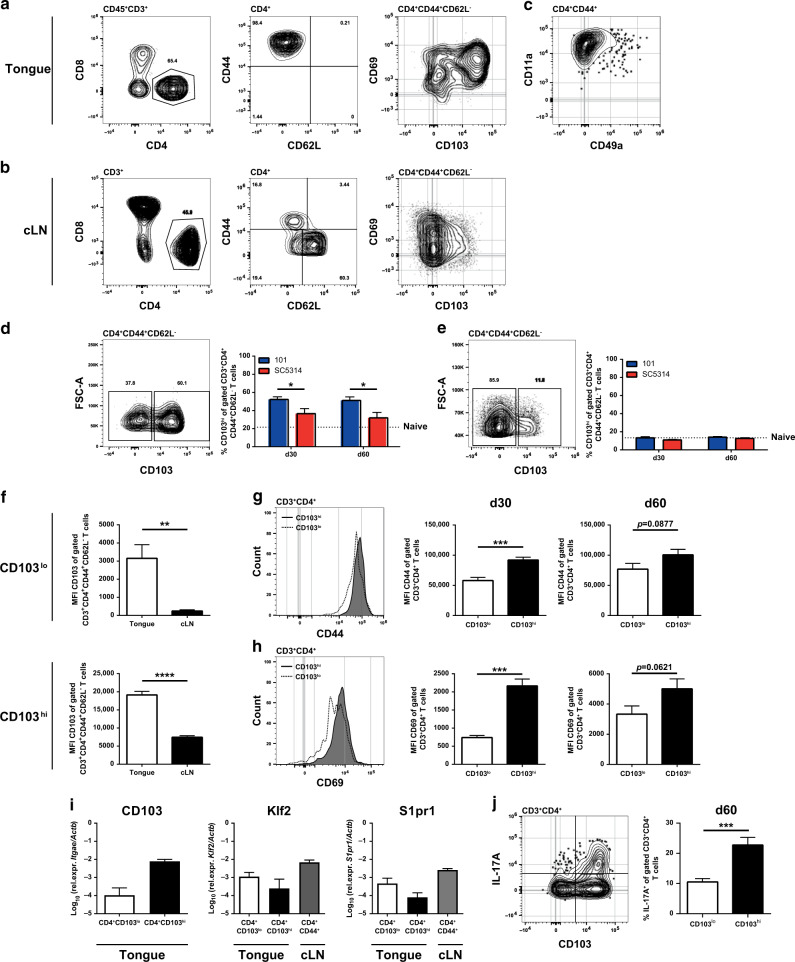
Tongue CD4^+^ T cells share phenotypic characteristics with T_RM_ cells. WT mice were infected sublingually with *C. albicans* strain 101 (blue) or strain SC5314 (red) for the indicated time points. **a–c** CD3^+^CD4^+^ T cells from the tongue (**a**, **c**) and the cervical lymph nodes (cLN, **b**) were analyzed by flow cytometry for CD44, CD62L, CD69, CD103, CD11a, and CD49a expression. Representative FACS plots show the situation in strain 101-infected mice on day 60 post infection. **d**, **e** CD44^+^CD62L^−^CD3^+^CD4^+^ T cells in the tongue (**d**) and the cervical lymph nodes (cLN, **e**) were divided into CD103^hi^ and CD103^lo^ subsets. Representative FACS plots (left) show the situation in strain 101-infected mice on day 60 post infection. Summary graphs (right) represent the mean + SEM of 6–13 individual mice per group pooled from 3–4 independent experiments. The dotted line represents the mean value determined in naive mice. **f** The mean fluorescence intensity (MFI) of the CD103 staining was analyzed in the CD103^lo^ (top) and CD103^hi^ (bottom) subsets of CD44^+^CD62L^−^CD3^+^CD4^+^ T cells in the tongue and the cervical lymph nodes (cLN). The MFI of the CD44 (**g**) and CD69 staining (**h**) was analyzed in the CD103^hi^ and CD103^lo^ subsets of tongue CD3^+^CD4^+^ T cells. Representative FACS plots (left) show the situation on day 60 post infection. Summary graphs (middle and right) represent the mean + SEM of 7–13 individual mice per group pooled from 2–3 independent experiments. **i** Tongue CD4^+^CD103^hi^ and CD4^+^CD103^lo^ T cell subsets and cervical lymph node (cLN) CD4^+^CD44^+^ T cells were sorted from strain 101-infected animals on day 60–90 post infection and *Klf2* and *S1pr1* transcripts were quantified by RT qPCR. *Itgae* transcripts (coding for CD103) were quantified in the tongue T_RM_ subsets as a control. Each bar represents the mean + SEM of 2–3 samples that were obtained by pooling the tongues or cervical lymph nodes from five mice each. **j** IL-17 production by tongue CD4^+^CD103^hi^ and CD4^+^CD103^lo^ T cell subsets was analyzed by flow cytometry on day 60 post infection and after ex vivo re-stimulation with PMA and ionomycin for 4 h in the presence of Brefeldin A. A representative FACS plot is shown on the left; quantification of the frequency of IL-17^+^CD3^+^CD4^+^ T cells in the tongue is shown on the right. Each bar represents the mean + SEM of five individual mice per group pooled from two independent experiments. Statistics were calculated using two-way ANOVA (**d**, **e**) or *t*-test (**f–h, j**), *p < 0.05, **p < 0.01, ***p < 0.001, ***p < 0.0001. See also Supplementary Figs. S3 and S4.

Next, we interrogated the nature of the CD4^+^ T cells in the tongue of *C. albicans*-colonized mice. The population expressed uniformly high levels of CD44^+^ and was negative for CD62L (Fig. 3a), a phenotype, corresponding to antigen-experienced effector/memory T cells, while in the cervical lymph nodes of the same animals, which were included in the analysis as a reference, only ~15% of all CD4^+^ T cells displayed this phenotype, as expected (Fig. 3b). Furthermore, the majority of the CD4^+^ tongue T cells expressed high levels of CD69 and CD103 (Fig. 3a), two markers characteristic for tissue-resident memory T cells.^54^ CD69 expression supports tissue retention by binding and sequestering the sphingosine-1-phosphate receptor 1 (S1pr1), which promotes tissue egress.^55^ CD103, the αE subunit of the integrin αEβ7, binds to E-cadherin expressed on epithelial cells and thereby mediates lymphocyte retention.^56^ Moreover, tongue Th cells in stably colonized mice expressed high levels of CD11a, and to some extent also CD49a, consistent with a T_RM_ phenotype (Fig. 3c).^54^ The frequency of CD103^hi^ T_RM_ cells remained high throughout the analysis period (Fig. 3d). In contrast, in mice that were only transiently exposed to *C. albicans*, CD103^hi^ T_RM_ cells made up only a minor part of the already rare CD4^+^ tongue T cells (Fig. 3d).

In contrast to the situation in the tongue, CD69^+^CD103^+^ T_RM_ cells were hardly detectable in the cervical lymph nodes of *C. albicans* strain 101-infected mice. Only a minute proportion of the CD44^+^CD62L^−^CD4^+^ T cells expressed these markers, irrespective of the fungal strain and the time point of analysis (Fig. 3b, e). The memory T cell subset that was most prominently increased in the cervical lymph nodes of stably colonized mice displayed a CD127^+^CD44^+^CD62L^−^ phenotype characteristic of effector memory T cells, while CD127^+^CD44^+^CD62^+^ central memory T cells and CD127^−^CD44^+^CD62L^−^ effector T cell subsets remained unchanged in comparison to naive mice or mice that had been transiently infected with strain SC5314 (Supplementary Fig. S3). Differences between the groups may appear small. However, it should be noted that antigen-specific T cells make up only a small proportion of all CD4^+^ T cells within the polyclonal T cell repertoire in the lymph nodes.

In the tongue of stably colonized mice, CD103 expression divided the CD4^+^ T_RM_ cells into two subsets (Fig. 3d). The CD103^hi^ and CD103^lo^ T_RM_ cell populations displayed higher expression levels of CD103 than their respective counterparts in the cervical lymph nodes (Fig. 3f). The average expression levels of CD44 and CD69 of tongue T_RM_ cells correlated with CD103 expression levels, i.e., the mean fluorescence intensity of both markers was higher in the CD103^hi^ compartment than in the CD103^lo^ compartment at any time point analyzed (Fig. 3g, h). Moreover, *Klf2* and *S1pr1* transcripts were expressed at lower levels by the CD103^hi^ subset of T_RM_ cells than in the CD103^lo^ subset and by CD44^+^ lymph node T cells (Fig. 3i), consistent with a role of Klf2 in controlling the expression of S1pr1, which is required for tissue egress.^57^ Finally, CD103^hi^ T_RM_ cells were the predominant source of IL-17 in the stably colonized tongue (Fig. 3j). Together, these results indicate that the accumulation and stable maintenance of T_RM_ cells at the site of fungal colonization is a hallmark of the IL-17-mediated immunosurveillance response to the commensal fungus.

T_RM_ cells in the tongue of stably colonized mice differ from TCRβ^+^ T cells, which contribute to the IL-17 response at the onset of acute OPC with strain SC5314,^41,42^ as the latter express lower levels of CD103, CD44, and CD11a at the cell surface as well as higher levels of Klf2 and S1pr1 transcripts (Supplementary Fig. S4).

### CD4^+^ T cells are located within the tongue parenchyma in stably colonized mice

**Fig. 4:**
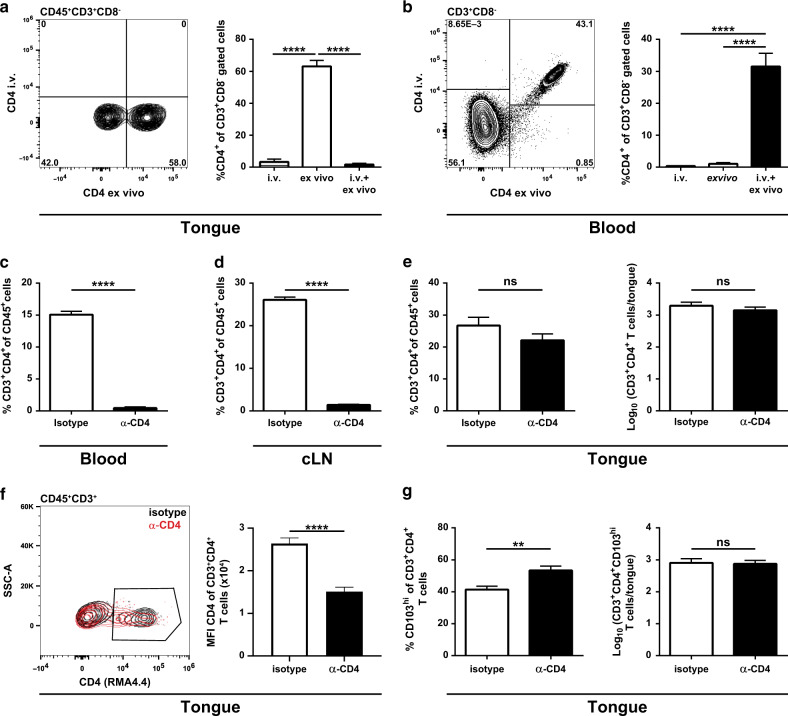
CD4^+^ T_RM_ cells are located within the tongue tissue and not in the vasculature. WT mice were infected sublingually with *C. albicans* strain 101. **a**, **b** Forty-two days post infection, mice were injected intravenously with fluorescently labeled anti-CD4 antibody (clone RMA4.4) 5 min prior to sacrifice. Tongue (**a**) and blood (**b**) CD3^+^CD8^−^ T cells were stained ex vivo with anti-CD4 (clone RMA4.5, conjugated to a different fluorochrome than the intravenously injected antibody). Representative FACS plots (left) and summary graphs (right) represent the percentages of intravenously labeled (i.v.), ex vivo stained and double-stained CD4^+^ T cells. **c**–**g** Twenty-one days post infection, mice were injected intraperitoneally with an anti-CD4 depleting antibody (clone GK1.5) or an isotype control for two consecutive days. Seven days later, the frequency of CD3^+^CD4^+^ T cells among CD45^+^ cells in the blood (**c**), cervical lymph nodes (cLN, **d**) and tongue (**e**) was determined after ex vivo staining with a non-competing anti-CD4 antibody (clone RMA4.4). In the tongue, the analysis was complemented with the absolute numbers of CD3^+^CD4^+^ T cells (**e**) and numbers and percentage of CD103^hi^ cells among CD3^+^CD4^+^ T cells (**g**). The median fluorescence intensity (MFI) of the CD4 (clone RMA4.4) staining on tongue CD45^+^ CD3^+^ T cells is shown in (**f**) with representative FACS plots (left) and a summary graph (right). Data are the mean + SEM of 7–10 mice per group pooled from two independent experiments. Statistics were calculated using one-way ANOVA (**a**, **b**) or *t*-test (**c**–**g**), **p < 0.01, ****p < 0.0001. See also Supplementary Fig. S5.

To verify whether tongue CD4^+^ T cells in stably colonized mice resided indeed within the tongue tissue and not in the vasculature, we selectively labeled T cells in the vasculature but not those within tissues by intravenous administration of a fluorochrome-conjugated anti-CD4 antibody five minutes prior to euthanasia. Blood and tongue T cells were stained ex vivo with a different clone of anti-CD4 to identify all CD4^+^ T cells, irrespective of whether they had access to the in vivo administered antibody or not, and analyzed them by flow cytometry. While all CD4^+^ T cells in the blood were stained with both antibody clones, the CD4^+^ T cells in the tongue were protected from the injected antibody, indicating that they were indeed localized within the tissue and had not been in contact with the circulation (Fig. 4a, b).

To provide further evidence for the tissue residency of the CD4^+^ T cells in the tongue of stably colonized mice, we administered a CD4-specific depleting antibody. To check the depletion efficiency, remaining CD4^+^ T cells were stained ex vivo with a non-competing antibody clone specific for CD4.^58^ 7 days after systemic administration of the antibody, the frequency of CD4^+^ T cells was strongly diminished in the blood and in the cervical lymph nodes (Fig. 4c, d), while CD8^+^ T cell frequencies were comparatively increased in comparison to mice treated with an isotype control antibody (Supplementary Fig. S5). In contrast, in the tongue, the number of CD4^+^ T cells remained nearly unchanged in CD4-depeleted animals compared to the isotype control group (Fig. 4e), although the median fluorescence intensity of the CD4 staining was somewhat reduced in depleting antibody-treated compared to isotype control-treated mice (Fig. 4f**)**. These findings suggested that the anti-CD4 antibody or cells mediating depletion of antibody-bound cells did not access the CD4^+^ T cells in the tongue parenchyma, even after the prolonged presence of the antibody in the circulation. This further indicated that during persistent colonization with *C. albicans*, CD4^+^ T cells are indeed tissue-resident and stably maintained in the tongue tissue independently of circulating CD4^+^ T cells. Interestingly, within the tongue CD4^+^ T cell compartment, we observed a shift in the ratio of CD103^hi^/CD103^lo^ T cells with a relative increase in the CD103^hi^ subset in the anti-CD4-treated group (Fig. 4g). This might suggest that the marginal depletion of CD4^+^ T cells that was still observed, affected preferentially the CD103^lo^ subset, which might be slightly more mobile in the epithelial tissue compared to their CD103^hi^ counterpart.

### Tongue CD4^+^ T cells in *C. albicans*-colonized mice are maintained independently of circulating T cells

**Fig. 5:**
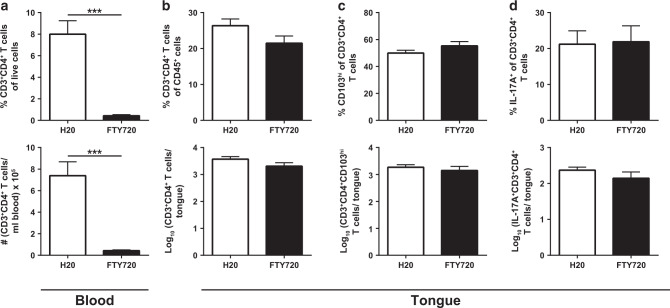
CD4^+^ T cells are maintained in the tongue of *C. albicans*-colonized mice independently of circulating T cells. WT mice were infected sublingually with *C. albicans* strain 101. 21 days post infection, FTY720 was administered in the drinking water for 3 weeks and CD3^+^CD4^+^ T cells in the blood and in the tongue were analyzed by flow cytometry. Frequencies (top) and absolute numbers (bottom) of CD3^+^CD4^+^ T cells in the blood (**a**) and in the tongue (**b**), respectively. **c** Frequency (top) and absolute numbers (bottom) of CD103^hi^CD3^+^CD4^+^ T cells in the tongue. **d** Frequency (top) and absolute numbers (bottom) of IL-17^+^CD3^+^CD4^+^ T cells in the tongue. Cytokine production was analyzed after re-stimulation with PMA and ionomycin for 4 h in the presence of Brefeldin A. Data are the mean + SEM of ten mice per group pooled from two independent experiments. Statistics were calculated using *t*-test, ***p < 0.001. See also Supplementary Fig. S6.

T_RM_ cells exhibit little or no potential to recirculate and are maintained largely independently of the circulating T cell pool.^59^ To examine, whether this held also true for the CD4^+^ T cells in the tongue of mice that are stably colonized with *C. albicans*, we employed FTY720 (Fingolimod), a S1pr1 antagonist, to block the egress of lymphocytes from secondary lymphoid organs, thereby preventing lymphocyte recirculation.^54^ Administration of FTY720 to stably colonized mice for 3 weeks resulted in a strong decrease in the frequency and total numbers of circulating CD4^+^ T cells in comparison to untreated controls (Fig. 5a). The same was also true for CD8^+^ T cells **(**Supplementary Fig. S6A). In contrast, in the tongue, CD4^+^ and CD8^+^ T cell frequencies and absolute numbers remained stable (Fig. 5b, Supplementary Fig. S6B). Within the CD4^+^ T cell population, the distribution of the CD103^hi^ and CD103^lo^ subsets remained unchanged upon treatment with FTY720 (Fig. 5c). Finally, also their capacity to produce IL-17 was unaffected by the administration of FTY720 (Fig. 5d). Together, these data indicate that CD4^+^ T cells are maintained in the *C. albicans*-colonized mucosal tissue independently of the circulation, and thereby meet another key criterion for T_RM_ cells.

### T_RM_ cells are sufficient to prevent overgrowth of commensal *C. albicans* in the oral mucosa

**Fig. 6:**
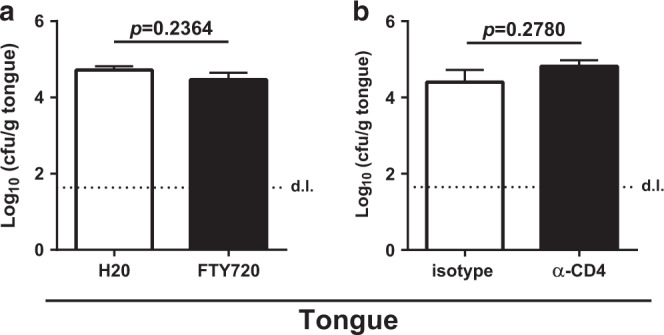
T_RM_ cells are sufficient for preventing overgrowth of commensal *C. albicans* in the oral mucosa. **a** WT mice were infected and treated with FTY720 as in Fig. 5. Plots show tongue cfu on day 42 post infection. **b** WT mice were infected and treated with depleting anti-CD4 antibody as in Fig. 4c–f. Plots show tongue cfu on day 7 after T cell depletion. Each bar represents the mean + SEM of 7–10 mice per group pooled from two independent experiments. Statistics were calculated using *t*-test. The dotted line represents the detection limit (d.l.). See also Supplementary Fig. S7.

Finally, we sought to determine whether T_RM_ cells in the *C. albicans*-colonized tongue were sufficient for maintaining commensalism and preventing fungal overgrowth. To this aim, we analyzed the fungal burden in the tongue of stably colonized mice that were treated for 3 weeks with FTY720, as described above (see Fig. 5), to prevent the supply of circulating T cells from the lymph nodes to the peripheral tissue. Indeed, FTY720-treatement did not affect *C. albicans* colonization loads (Fig. 6a), supporting the notion that T_RM_ cells mediate antifungal control independently of the circulatory T cell compartment.

Moreover, we assessed the fungal burden in *C. albicans*-colonized mice, in which all circulating CD4^+^ T cells, but not their tissue-resident counterparts, were depleted by means of an anti-CD4 antibody (see Fig. 4c–f). Importantly, the colonization load in the tongue was not significantly affected by the depletion of CD4^+^ T cells in the blood and cervical lymph nodes (Fig. 6b), indicating that the *C. albicans*-specific CD4^+^ T cells retained in the tongue after anti-CD4 administration were sufficient to keep fungal growth in check, independently of the circulating antifungal CD4^+^ T cell pool. The situation in both of these settings was thus markedly distinct from what we observed in *Rag1*^*−/−*^ and *TCRbd*^*−/−*^ mice that are completely devoid of T cells, where fungal growth was out of control (see Supplementary Fig. S1). Together, these results provide evidence that bona fide T_RM_ cells in the *C. albicans*-colonized mucosa provide local surveillance of fungal commensalism.

### T_RM_ cells level out fluctuations in the *C. albicans* colonization load

We hypothesized that the maintenance of a high frequency of *C. albicans*-specific T_RM_ cells in stably colonized mice might confer an advantage by creating a state of alert that ensures a rapid response to fluctuations in the fungal load as they naturally occur.^60,61^ To simulate such oscillations in the fungal load in our experimental model, we chose to superinfect stably colonized mice with an additional inoculum of *C. albicans* (Supplementary Fig. S7A). Within 4 days after superinfection, we observed a significant increase in IL-17-secreting T_RM_ cells in the tongue compared to stably colonized mice that were not superinfected (Supplementary Fig. S7B–D). Of note, the enhanced Th17 response observed during the superinfection correlated with improved fungal control (Supplementary Fig. S7E). The tongue fungal load in superinfected mice was lower than that of non-primed mice, which were infected for just 4 days and even lower than in persistently colonized animals that were not superinfected (Supplementary Fig. S7E). Together, these results emphasize the relevance of T_RM_ cells in the colonized tissue to level fluctuations in colonization load and to retain homeostatic levels of commensal *C. albicans*.

### Tongue T_RM_ cells provide enhanced fungal control after cleared infection and secondary fungal exposure

**Fig. 7:**
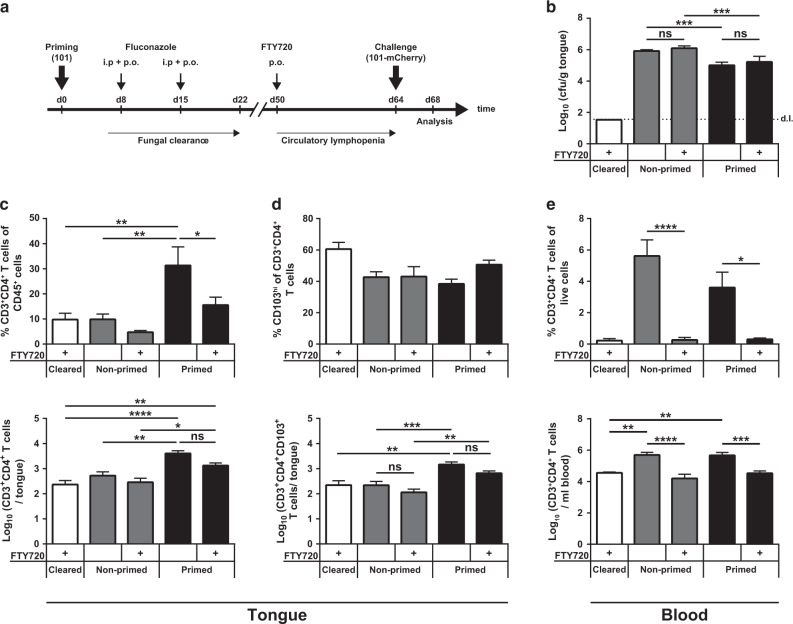
Tongue T_RM_ cells also provide protection against transient infection during secondary exposure. WT mice were infected sublingually with *C. albicans* strain 101 and treated with fluconazole for 2 weeks starting from day 8 post infection. Starting from day 42 post infection, mice were or were not treated with FT720, as indicated, and were re-infected 14 days later with strain 101-mCherry for 4 days. As a control, a group of naive mice was included that did not receive the first infection with strain 101 (non-primed). Moreover, a group of mice was included, which was transiently infected with strain 101 and then treated with fluconazole but did not receive the second infection with strain 101-mCherry (cleared), as indicated. Four days after re-infection, fungal burden and T cells were analyzed in the tongue. **a** Schematic outline of the experiment. **b** Tongue cfu on day 4 post infection in the indicated groups. The dotted line represents the detection limit (d.l.). The frequencies (top) and absolute numbers (bottom) of CD3^+^CD4^+^ T cells in the tongue (**c**) and in the blood (**e**) and of CD103^hi^CD3^+^CD4^+^ T cells in the tongue (**d**) were analyzed by flow cytometry. Each bar represents the mean + SEM of 6–8 mice per group pooled from two independent experiments. Statistics were calculated using or one-way ANOVA, *p < 0.05, **p < 0.01, ***p < 0.001, ****p < 0.0001. See also Supplementary Fig. S8.

The function of T_RM_ cells has been studied before in various infection models with organisms that are readily cleared in mice, including a model of epicutaneous candidiasis with strain SC5314 where T_RM_ cells were found to significantly improve pathogen control during re-infection with the same organism.^62^ In Fig. 3, we observed that a small population of tongue T_RM_ cells was also induced during transient OPC. Therefore, we questioned whether T_RM_ cells contribute to or possibly even suffice for fungal control during recurrent candidiasis in the oral mucosa as during persistent colonization. We employed the model of transient OPC with strain 101 as in Fig. 2c, in which we eliminated the fungus on day 8 after the primary infection by administration of fluconazole (Fig. 7a). As expected, transient exposure of mice to *C. albicans* resulted also in the generation of circulatory T cell memory as evidenced by an enhanced Th17 response in the cervical lymph nodes upon re-infection (Supplementary Fig. S8A) and an increased proliferation of T_EM_ cells (Supplementary Fig. S8B, C). We speculated that these circulatory memory T cells possibly contribute to local fungal control in the oral mucosa during re-infection. Therefore, to uncouple the involvement of T_RM_ cells in the tongue from circulatory memory T cells, we made use of FTY720 treatment. FTY720 was administered to mice that had or had not been transiently exposed to *C. albicans* strain 101 to induce circulatory lymphopenia (Fig. 7a). Importantly, while pre-existing antifungal memory conferred enhanced fungal control, FTY720 treatment had no impact on the tongue fungal load in primed mice (Fig. 7b), indicating that T_RM_ cells in the tongue were sufficient for optimal fungal control and circulatory memory T cells were not required. Consistent with this, we observed a stark increase in the number of tongue CD4^+^ T cells upon re-infection (Fig. 7d), most of which expressed high levels of CD103 (Fig. 7e), and this was largely independent of FTY720. In contrast, blood CD4^+^ T cells were markedly decreased upon FTY720 treatment (Fig. 7c), as expected. Collectively, both in transiently infected and in stably colonized mice, a population of T_RM_ cells persists locally in the tongue and efficiently controls the fungus to either prevent overgrowth during recurrent infection or to control persistent fungus during commensalism, respectively.

## Discussion

Fungi are increasingly recognized as integral part of the microbiota. Their interaction with the host can have manifold consequences, as exemplified by *C. albicans*, which confers host-beneficial properties but at the same time bears a risk for the host through its potential to cause infection and/or to promote immunopathology.^3^ Tight control by the immune system is essential to prevent dysbiosis. IL-17-mediated immunity and especially Th17 cells are indispensable for the control of *C. albicans* in mucosal barrier tissues. However, the Th17 response to *C. albicans* during homeostasis remains poorly characterized. In the past, most mechanistic studies on antifungal T cell immunity employed models of acute and transient infection in mice. In this study, we made use of a novel experimental model of persistent oral colonization and found that *C. albicans*-induced T cells in the stably colonized tongue exhibit phenotypic and functional properties of T_RM_ cells, including high expression of CD103, CD69, and CD11a, low expression of Klf2 and S1pr1, localization within the tissue and maintenance independently of the circulation. Importantly, we found that these T_RM_ cells are responsible for mediating immunosurveillance of the fungus and to maintain stable commensalism.

Antigen-specific Th17 cells are efficiently primed in response to *C. albicans*, irrespective of the strain virulence.^45^ T cell activation and differentiation in experimentally infected mice is comparable between the low-virulent strain 101 and the highly virulent strain SC5314, although immediate innate immunity and the degree of inflammation induced at the site of infection differ greatly between the two strains.^45^ Differences in the Th17 response become apparent only later during infection. While the *C. albicans*-specific T cell pool rapidly contracts after resolution of acute OPC, Th17 cells are stably maintained at high numbers in the oral mucosa during persistent colonization. Reminiscent of what was recently reported in a model of intestinal colonization with *C. albicans,*^18^ the maintenance of a large *C. albicans*-specific T cell population depends on the continuous presence of the fungus. The same was also observed in various models of bacterial and parasitic infections.^63–65^ The fact that fungus-specific T cells are only maintained at high numbers if they are needed, may represent a means to prevent immunopathology, as *C. albicans*-specific T cells can also bear a pathogenic potential.^18,19^

The Th17 response is stably maintained in colonized mice without IFN-γ- or GM-CSF-producing T cells appearing over time. This is consistent with the absence of inflammation in colonized mice, even under conditions of reduced immune regulation, such as regulatory T cells or IL-10 deficiency.^48^ In contrast, dysregulation of the Th17 response results in uncontrolled fungal growth. Together, our new model of *C. albicans* colonization closely mimics the situation of *C. albicans* commensalism in humans and therefore represents a suitable model for studying the immune mechanisms that regulate homeostasis at the fungus-host interface. Importantly and in contrast to other currently available models of fungal colonization in mice, such as the model of gastrointestinal colonization,^66^ our model does not depend on the pretreatment of mice with antibiotics or immunosuppressants. Furthermore, our model recapitulates the situation in humans regarding the quality of the Th17 response. *C. albicans*-specific Th17 cells can be readily detected in the blood of all individuals that have been exposed to the fungus during the course of their life. These human circulating T cells have a memory phenotype,^67,68^ consistent with the T_EM_ cells that we observed in the lymph nodes of stably colonized mice. In contrast, *C. albicans*-specific memory T cells in the mucosa, where *C. albicans* effectively resides, belong to the T_RM_ cell subset. T_RM_ cells that react to *C. albicans* have also been identified in the normal skin of healthy individuals.^62^

T_RM_ cells are strategically located in epithelial barrier tissues such as the skin, the lung, and the gastrointestinal and female reproductive tract. Phenotypically, they are characterized by the constitutive expression of the tissue retention markers CD69 and CD103.^54,59,69^ The majority of all studies on T_RM_ cells to date have focused on CD8^+^ T_RM_ cells, but CD4^+^ T_RM_ cells share the key hallmarks of this specialized subset of memory T cells.^70^ Our study is the first to report on CD4^+^ T_RM_ cells in the oral mucosa. Tongue CD4^+^ T_RM_ cells share all the phenotypic and functional characteristics that have been established for T_RM_ cells in other tissues.

Functionally, T_RM_ cells are poised for rapid local effector function, independently of the circulating pool of T cells. Their functional relevance has been studied in infection models with various pathogens that are swiftly cleared from the murine host. Evidence for a protective role of T_RM_ cells in these models was established under settings of re-call infections.^54^ These examples include a model of acute *C. albicans* skin infection with strain SC5314.^62^ We confirmed these published data in our study using a setting of transient OPC. A host-protective role of T_RM_ cells has also been evidenced in the context of chronic infections with pathogenic viruses, such as HSV and CMV.^71^ In human tissues, CD4^+^ or CD8^+^ T_RM_ cells have been identified, including those specific to CMV, RSV, Epstein-Barr virus, HSV, HBV, HIV, and IAV^54^ and correlations have been made between increased populations of virus-specific T_RM_ cells and enhanced viral control.^72–75^ Moreover, growing evidence points toward a role for T_RM_ cells in inflammatory disorders and autoimmunity, both in humans and mice.^69^

Keeping in check the myriads of (normally harmless) microbes to which we are constantly exposed is probably the most profound challenge for the immune system. The lack of tight control of the microbiota results in microbial overgrowth at the epithelial barriers and microbial invasion of internal organs, which are usually sterile. Therefore, effective immune mechanisms are required to ensure a peaceful cohabitation with commensal organisms, on which we strongly depend for important physiological functions. Homeostatic immunity is a highly complex process that involves cellular and humoral components of the immune system. Our study adds T_RM_ cells as a central component of effective long-term surveillance of commensal organisms, as exemplified by persistent *C. albicans* colonization in the oral mucosa. Based on our results, we postulate that regulating homeostasis of the microbiota may represent the most fundamental role of T_RM_ cells in addition to all other scenarios, in which they have been implicated. Although our study is limited to *C. albicans*, a common and well documented inhabitant of the oro-gastrointestinal and female reproductive tract, T_RM_ cells are likely implicated more generally in the control of other fungal and bacterial commensals.

## Materials and methods

### Reagents

All reagents, antibodies, mouse and fungal strains, instruments, and software used in this study are listed in Supplementary Table S1. Further information and requests for resources and reagents should be directed to and will be fulfilled by the lead contact S.L.L. The DC^1940^ cell line (University of Lausanne) was obtained via an MTA between the University of Zürich and the respective entity.

### Ethic statement

All mouse experiments in this study were conducted in strict accordance with the guidelines of the Swiss Animals Protection Law and were performed under the protocols approved by the Veterinary office of the Canton of Zürich, Switzerland (license number 201/2012, 183/2015 and 166/2018). All efforts were made to minimize suffering and ensure the highest ethical and humane standards according to the 3R principles.

### Animals

WT C57BL/6j mice were purchased by Janvier Elevage. *Rag1*^−/−,^^76^ *TCRbd*^−/−,^^77,78^ *Il17af*^−/−,^^79^ Tg(TcraTcrb)603Biat (Hector),^49^ *Il23r*^*GFP/GFP*^ mice^80^ as well as congenic CD45.1^+^ and CD45.1^+^CD45.2^+^ WT mice were bred and maintained at the Institute of Laboratory Animals Science (LASC), University of Zürich. All mice were on the C57BL/6 background. The animals were kept in specific pathogen-free conditions and used at 6–15 weeks of age in sex- and age-matched groups. Infected and uninfected animals were kept separately to avoid cross-contamination. Female as well as male mice were used for experiments.

### Fungal strains

*C. albicans* strains SC5314^81^ and 101^45^ were grown in YPD medium at 30 °C and 180 rpm for 15–18 h. 101-mCherry was generated by transformation of strain 101 with a plasmid pADH-Cherry-SAT1^82^ digested with *Bam*HI, which introduces the plasmid into the *ADHI* locus. Transformation was performed as described by a lithium acetate method.^83^ Selection of transformants was carried out on nourseothricin-containing YPD plates (200 µg/ml).

### Murine OPC model

Mice were infected sublingually with 2.5 × 10^6^ *C. albicans* yeast cells as described,^84^ without immunosuppression. In some experiments, mice were treated with Fluconazole (400 µg/mouse i.p. on day 7–8 post infection followed by administration of 0.2 mg/ml Fluconazole in the drinking water). Fungal clearance was checked 7 days after the initial treatment by plating fecal material on YPD plates. Secondary infections (re-infection or superinfection) were done  ~60 days after the primary infection using *C. albicans* strain 101-mCherry to discriminate the fungus from the primary and secondary infection.

### Determination of the fungal burden

For determination of the fungal burden, the tongue of euthanized animals was removed, homogenized in sterile 0.05% NP40 in H_2_O for 3 min at 25 Hz using a Tissue Lyzer (Qiagen) and serial dilutions were plated on YPD agar containing 100 µg/ml Ampicillin.

### Histology

For histology, tissue was fixed in 4% PBS-buffered paraformaldehyde overnight and embedded in paraffin. Sagittal sections (9 µm) were stained with Periodic-acidic Schiff reagent and counterstained with Haematoxilin and mounted with Pertex (Biosystem) according to standard protocols. Images were acquired with a digital slide scanner (NanoZoomer 2.0-HT, Hamamatsu) and analyzed with NDP.view2.

### CD4^+^ T cell depletion

Mice were injected intraperitoneally with 500 µg of anti-CD4 antibody (clone GK1.5, BioXCell) or rat IgG2b isotype control (BioXCell) on day 21 and again on day 22 post infection. Mice were analyzed 7 days after antibody administration. anti-CD4 (clonse RMA4.4) was used to monitor the efficiency of T cell depletion by flow cytometry.

### FTY720 treatment

Mice were treated with 5 µg/ml FTY720 (Selleckchem) in the drinking water. The depletion efficiency was monitored by quantification of CD4^+^ T cells in the blood on the day of analysis.

### Isolation of lymph node cells and ex vivo T cell re-stimulation

Cervical lymph nodes were removed, and single cell suspensions were prepared by digestion with DNase I (2.4 mg/ml, Roche) and Collagenase I (2.4 mg/ml, Invitrogen) in PBS for 15 min at 37 °C. For inducing cytokine production by primed T cells, 10^6^ cervical lymph node cells were re-stimulated for 6 h with 1 × 10^5^ DC^1940^ cells^85^ that were pulsed with 2.5 × 10^5^/ml heat-killed *C. albicans*. Unpulsed DC^1940^ cells served as negative control. Brefeldin A (10 μg/ml, AppliChem) was added for the last 5 h to inhibit the secretory pathway.

### Isolation of tongue cells and ex vivo T cell re-stimulation

Mice were perfused by injection of PBS into the right heart ventricle prior to removing the tongue. Tongues were cut in half and the underlying muscle tissue was carefully removed using a scalpel. The remaining tongue tissue was cut into small pieces and digested with DNase I (2.4 mg/ml) and Collagenase IV (2.4 mg/ml) in PBS for 45 min at 37 °C. Single cell suspensions were obtained by passing the digested tissue through a 70 μm strainer using ice cold PBS supplemented with 1% FCS and 2 mM EDTA and then stained for flow cytometry. For inducing cytokine production by tongue T cells, cells were stimulated with phorbol 12-myristate 13-acetate (PMA, 50 ng/ml, Sigma-Aldrich) and Ionomycin (500 ng/ml, Sigma-Aldrich) for 4 h in the presence of Brefeldin A (10 μg/ml) at 37 °C.

### Flow cytometry

Single cell suspensions of tongue and lymph nodes were stained in PBS supplemented with 1% FSC, 5 mM EDTA and 0.02% NaN_3_. LIVE/DEAD Near IR stain (Life Technologies) was used for exclusion of dead cells. The antibodies for surface and intracellular cytokine staining are listed in Supplementary Table S1. For intracellular cytokine staining, cells were fixed and permeabilized using BD Cytofix/Cytoperm reagent (BD Bioscience) and subsequently incubated in Perm/Wash buffer (BD Bioscience). All extracellular and intracellular staining steps were carried out on ice. For intranuclear Ki-67 staining, cells were fixed and permeabilized for 40 min at RT using the Foxp3 Staining Buffer Set (eBioscience) and subsequently stained for 40 min at RT in Permeabilization buffer (eBioscience). Cells were acquired on a FACS Gallios (Beckman Coulter) or a SP6800 Spectral Analyzer (Sony). Data were analyzed with FlowJo software (FlowJo LLC). The gating of the flow cytometric data was performed according to the guidelines for the use of flow cytometry and cell sorting in immunological studies,^86^ including pre-gating on viable and single cells for analysis. Absolute cell numbers of lymphocyte populations were calculated based on a defined number of counting beads (BD Bioscience, Calibrite Beads), which were added to the samples before flow cytometric acquisition.

### FACS sorting, RNA isolation and RT qPCR

For sorting cells from the tongue and cervical lymph nodes, single cell suspensions were stained in PBS, supplemented with 1% FSC and 5 mM EDTA, with a viability marker and antibodies directed against CD45, TCR-β, CD3, CD4, CD44, and CD103. Using a FACS Aria III (BD Biosciences), 400–2500 target cells per defined population were sorted per well of a 96-well plate (Eppendorf) containing RLT Plus RNeasy^®^ lysis buffer (Qiagen). Lysates were snap-frozen and stored at −80 °C until further processing. Whole-transcriptome amplification was performed following the Smart-Seq2 protocol^87^ and according to ref. ^41^. RT-qPCR was performed using SYBR Green (Roche) and a QuantStudio 7 Flex (Life Technology) instrument. The primers are listed in Supplementary Table S1^34,57,88^. All RT-qPCR assays were performed in duplicates and the relative expression (rel. expr.) of each gene was determined after normalization to *Actb* transcript levels.

### Intravascular CD4 staining

Mice were injected intravenously with 5 µg of FITC- or BV605-conjugated anti-CD4 antibody (clone RMA4.4) in 200 μl of PBS 5 min prior to euthanasia. Blood was collected prior to perfusion of the mice and removal of the tongue. Ex vivo staining of T cells was carried out using anti-CD4 antibody (clone RMA4.5).

### Adoptive transfer of *C. albicans*-primed polyclonal CD4^+^ T cells

WT mice were infected sublingually for 8 days with *C. albicans* strain 101. Cervical lymph nodes were processed as described above and CD4^+^ T cells were enriched to high purity using anti-CD4 microbeads following the manufacturer’s instructions (Miltenyi Biotec). A total of 10^6^ CD4^+^ T cells were injected intravenously into *Rag1*^*−/−*^ mice, which were infected sublingually with *C. albicans* strain 101 on the following day.

### Adoptive transfer of *C. albicans*-specific Hector CD4^+^ T cells

CD4^+^ T cells were first enriched from spleens of naive TCR-transgenic Hector mice^49^ with anti-CD4 microbeads (Miltenyi Biotec) following the manufacturer’s instructions and then stained with a viability marker and antibodies directed against CD3, CD4, CD90, CD44, and TCRVα2 and sorted on a FACS Aria II Cell Sorter (BD Biosciences). A total of 10^6^ CD4^+^TCRVα2^+^CD44^−^ Hector CD4^+^ T cells were injected intravenously into *Rag1*^*−/−*^ mice 1 day prior to sublingual infection with *C. albicans* strain 101. Hector T cells were identified by gating on CD3^+^CD4^+^CD90.1^+^TCRVα2^+^ cells.

### Chimeras

For generation of mixed bone marrow chimeras, CD45.1^+^CD45.2^+^ WT recipient mice were irradiated twice with a dose of 5.5 Gy at an interval of 12 h. Next, bone marrow from CD45.1^+^ WT and CD45.2^+^ *Il23r*^*GFP/GFP*^ donor mice was collected, washed twice with PBS under sterile conditions, mixed at a 1:1 ratio and injected intravenously into irradiated recipient mice 18 h after the second irradiation. Mice were treated with Borgal^®^ (MSD Animal Health GmbH) p.o. for the first 2 weeks of a total of a 8 week reconstitution period.

### Quantification and statistical analysis

Statistical significance was determined by unpaired Student’s *t* test with Welch’s correction or one- or two-way ANOVA with the Dunnet’s or Tukey’s multiple comparison test, as appropriate, using GraphPad Prism software. Data displayed on a logarithmic scale were log-transformed before statistical analysis. Outlier calculation was performed using the ROUT method. Significance is indicated as follows: *p < 0.05; **p < 0.01; ***p < 0.001; ****p < 0.0001.

## Supplementary information

Supplementary Information

## Data Availability

All raw data and metadata linked to this study are available on Zenodo (10.5281/zenodo.3933969).
