# Supplementary material for: Tissue-resident memory Th17 cells maintain stable fungal commensalism in the oral mucosa
Source: Mucosal Immunol. 2020 Jul 27;14(2):455–67. doi: 10.1038/s41385-020-0327-1 (PMC7946631; doi:10.1038/s41385-020-0327-1)
Supplement: Supplementary file 1 — Supplementary Information [file 41385_2020_327_MOESM1_ESM.pdf]

Figure S1

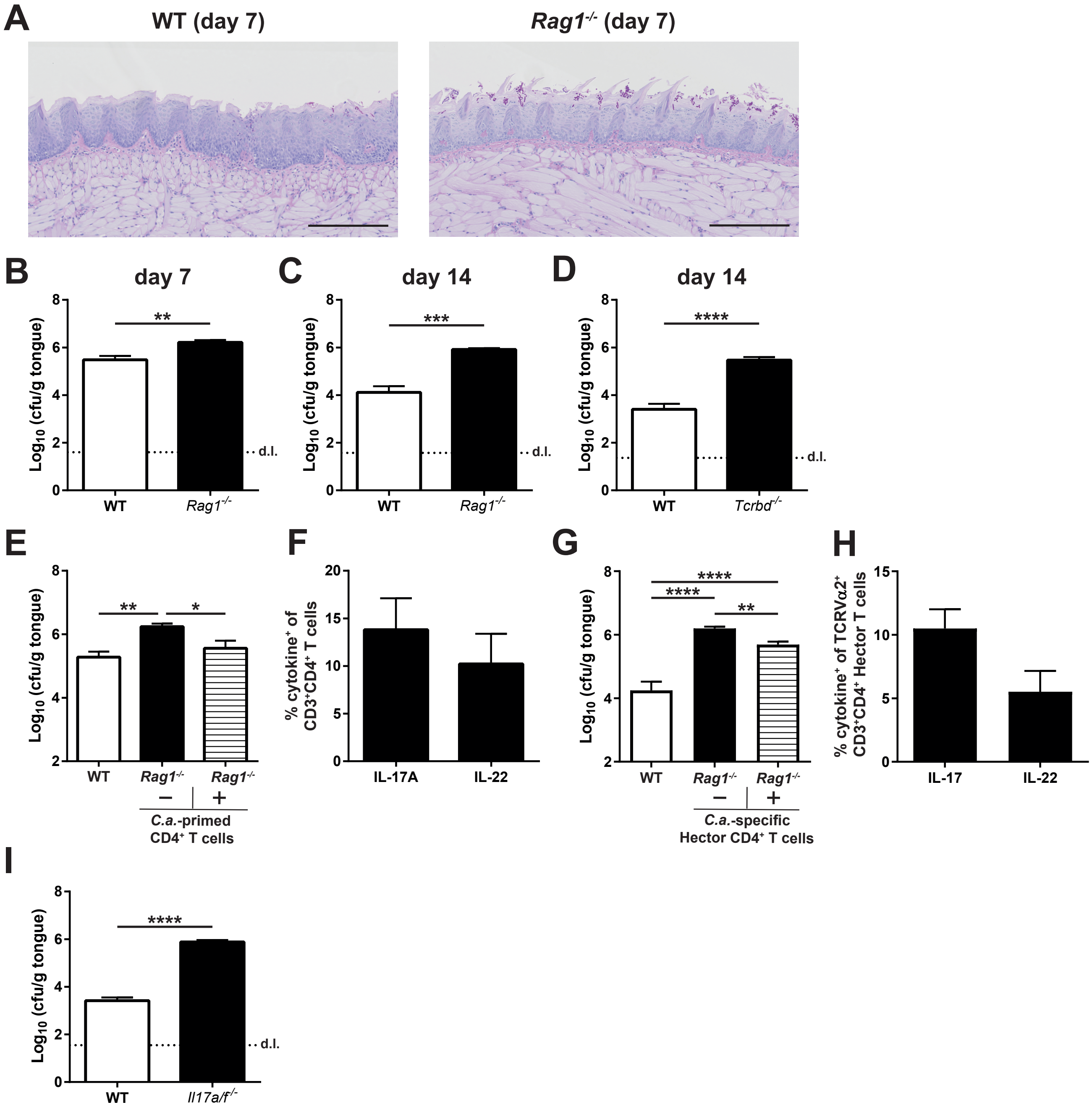

Figure S1 (related to Figure 1): T cells are required for preventing fungal overgrowth during *C. albicans* colonization of the oral mucosa with strain 101. WT and *Rag1*-/-, *Tcrbd*-/- or *Il17a/f*-/- mice were infected sublingually with *C. albicans* strain 101. (A) Representative images of sagittal tongue sections stained with PAS on day 7 post-infection. Scale bars: 250 µm. (B-D) The fungal burden in the tongue was determined by plating tissue homogenates on YPD agar on day 7 (B) or day 14 (C, D) post-infection. Data are the mean + SEM of 6-9 individual mice per group pooled from 2 independent experiments per time point. (E-H) *Rag1*-/-mice received an adoptive transfer of *C. albicans*-primed polyclonal CD4+ T cells (E) or naive *C. albicans*-specific TCR transgenic Hector CD4+ T cells (G) one day prior to infection with *C. albicans* strain 101, as indicated. The fungal load was determined as in B-D on day 7 (E) and day 14 (G) post-infection. Cervical lymph node cells were re-stimulated with *C. albicans*-pulsed DC1940 cells for 5 hours in the presence of Brefeldin A and IL-17A and IL-22 production was analyzed by intracellular cytokine staining and flow cytometry (F, H). Data are the frequency of cytokine producing CD3+CD4+ cells. Data are the mean + SEM of 6-14 (E, G) or 5-11 (F, H) individual mice per group pooled from 3-4 independent experiments. (I) The tongue fungal burden was determined as in B-D on day 28 post-infection. Data are the mean + SEM of 7-8 individual mice per group pooled from 2 independent experiments. The dotted line represents the detection limit (d.l.). Statistics were calculated using t-test (B-D, I) or one-way ANOVA (E, G), p*<0.05, **< 0.01, p***< 0.001, p****< 0.0001.

Figure S2

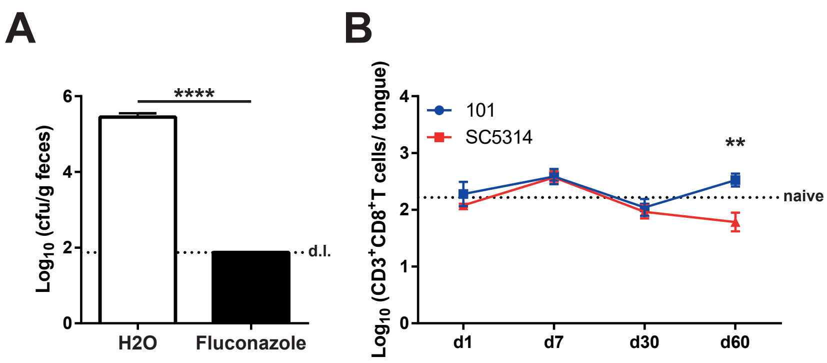

Figure S2 (related to Figure 2). Stable colonization of mice with *C. albicans* results in a persistent population of Th17 cells but not CD8+ T cells in the tongue. (A) WT mice were infected sublingually with *C. albicans* strain 101. On day 8 post-infection, mice were or were not treated with fluconazole for 14 days, as indicated. Clearance of *C. albicans* in the fluconazole-treated group was verified by plating fecal homogenates on YPD agar. d.l., detection limit. Data are the mean + SEM from 7- individual mice per group pooled from 2 independent experiments. (B) WT mice were infected sublingually with *C. albicans* strain 101 (blue) or strain SC5314 (red), respectively, for the indicated periods of time. CD3+CD8+ T cells in the tongue were quantified at the indicated time points post-infection using flow cytometry and the gating strategy shown in Figure 2A. Each symbol represents the mean + SEM of 6-13 individual mice per group pooled from 3 independent experiments. The dotted line represents the mean value determined in naïve mice. Statistics were calculated using t-test (A) or 2-way ANOVA (B), p**<0.01, p****< 0.0001.

Figure S3

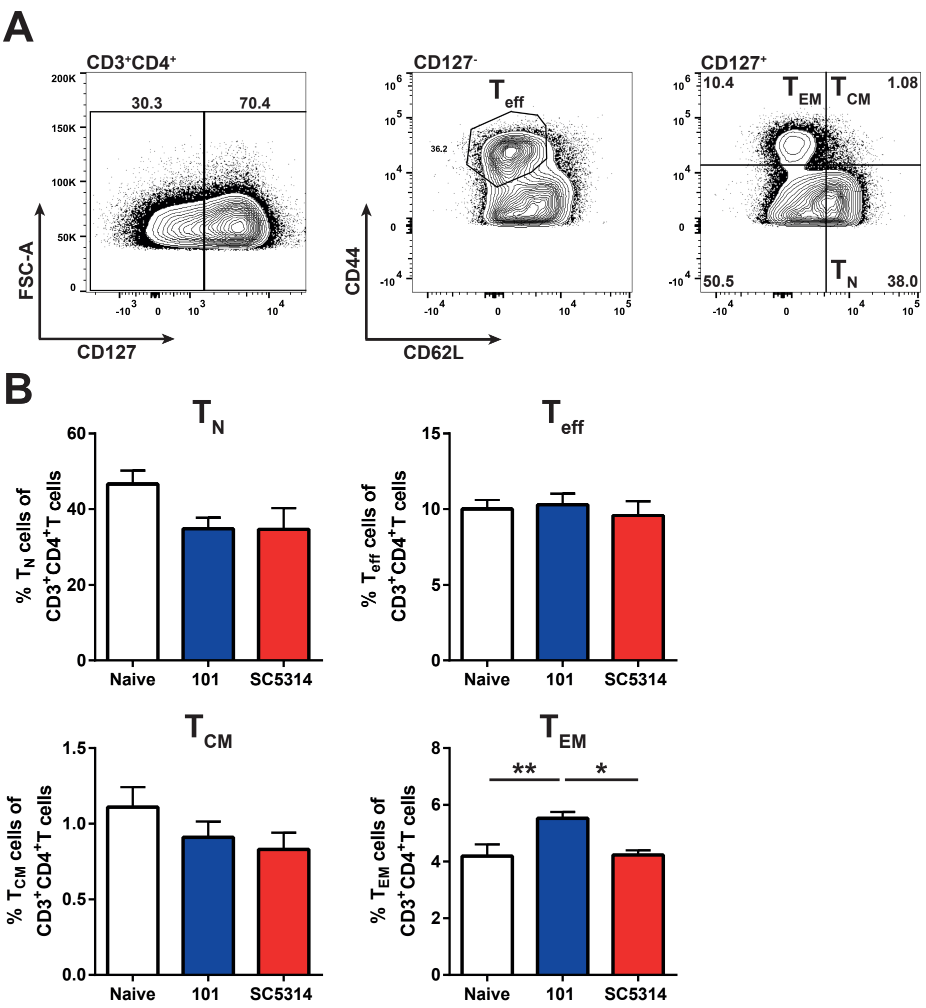

Figure S3 (related to Figure 3). ***C. albicans*** **colonization induces CD127+CD44+CD62L-CD4+ effector memory T cells in the cervical lymph nodes.** WT mice were infected sublingually with *C. albicans* strain 101 (blue) or SC5314 (red) and the cervical lymph nodes were analyzed for CD3+CD4+ T cell subsets. (A) Representative FACS plots showing the gating of CD127+CD44-CD62L+ naïve T cells (TN), CD127-CD44+CD62L- effector T cells (Teff), CD127+CD44+CD62L+ central memory T cells (TCM) and CD127+CD44+CD62L- effector memory T cells (TEM). (B) Quantification of naïve T, Teff, TCM and TEM cell frequencies among CD3+CD4+ T cells on day 60 post-infection. Data are the mean + SEM from 7-11 individual mice per group pooled from 2-3 independent experiments. Statistics were calculated using one-way ANOVA, p*< 0.05, p**<0.01..

Figure S4

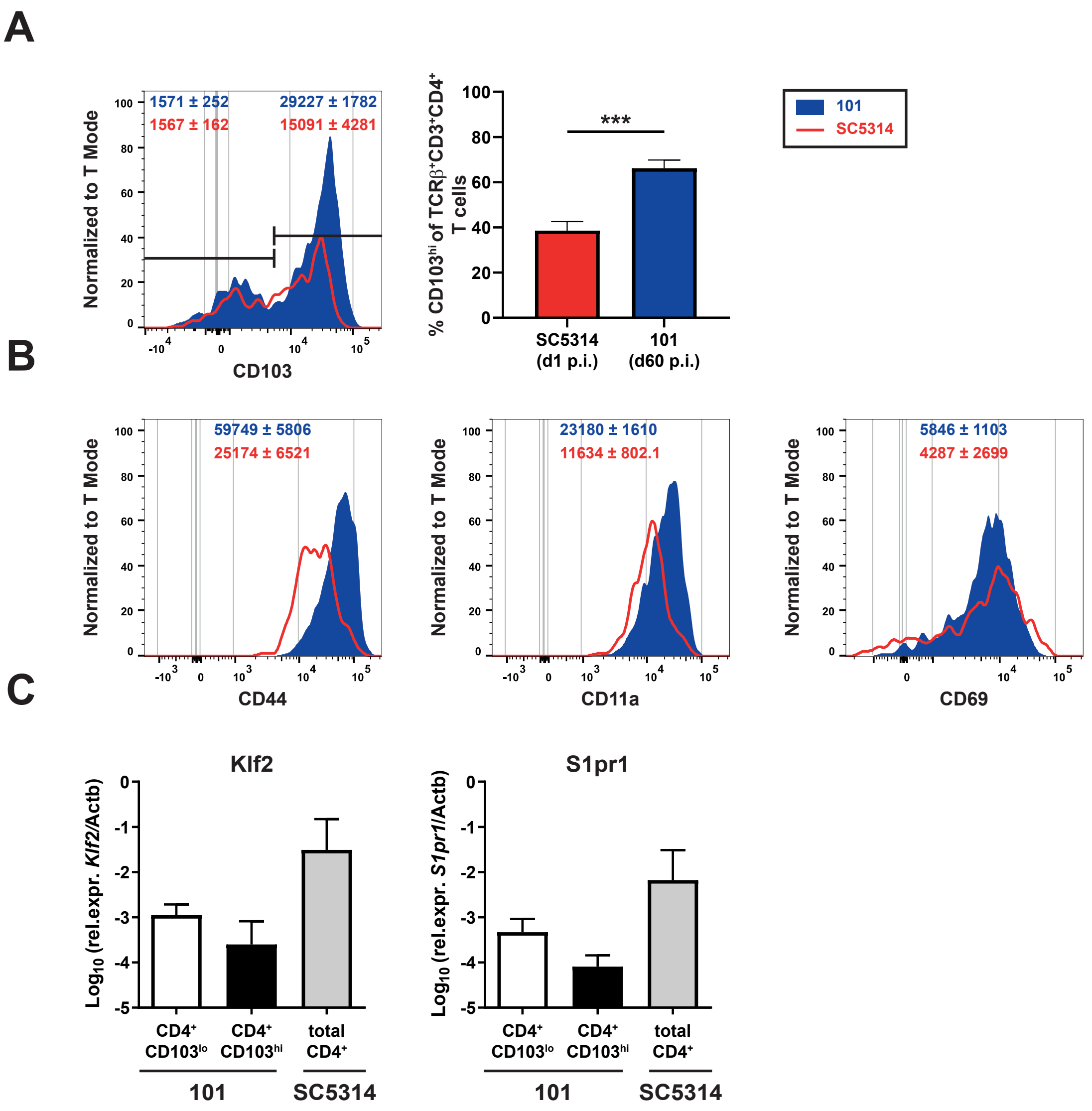

Figure S4 (related to Figure 3). TRM cells in the tongue of stably colonized mice are distinct from TCRβ+ T cells that respond to acute OPC. WT mice were infected sublingually with *C. albicans* strain 101 for 60-90 days (blue) or strain SC5314 for 1 day (red). (A) The Median fluorescence intensity (MFI) of CD103 staining (left) and the frequency (right) of the CD103hi and CD103lo TCRβ+CD3+CD4+ subsets in the tongue was determined. Numbers represent the mean + SD of 4-5 individual mice per group from one representative out of two independent experiments (left) and the mean + SEM from 3-5 individual mice per group pooled from 2 independent experiments (right). (B) MFI of CD44, CD11a and CD69 staining for the total TCRβ+CD3+CD4+ population was determined in the tongues of infected mice. Numbers represent the mean + SD of 4-5 individual mice per group from one representative out of two independent experiments. (C) Klf2 and S1pr1 transcripts were quantified by RT-qPCR in TCRβ+CD3+CD4+CD103hi and TCRβ+CD3+CD4+CD103lo T cell subsets sorted from the tongue of strain 101-infected mice and in total TCRβ+CD3+CD4+ cells sorted from the tongue of strain SC5314-infected animals. Data from strain 101-infected mice are the same as those shown in Fig 3I. Each bar represents the mean + SEM from 2-3 samples that were obtained by pooling the tongues of 4-5 mice each. Statistics were calculated using t-test (A) p***< 0.001.

Figure S5

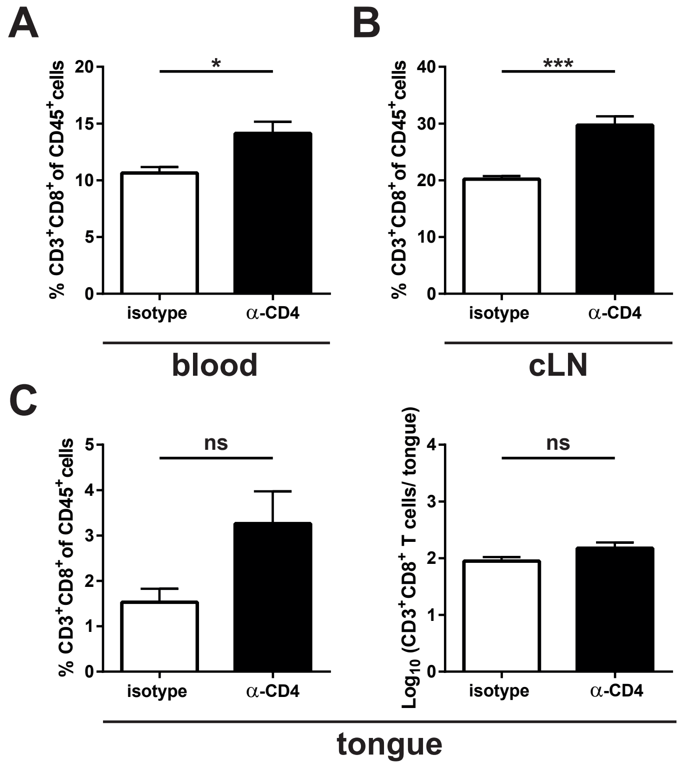

Figure S5 (related to Figure 4). CD4+ T cell depletion results in a relative increase in CD8+ T cells in the tongue. WT mice were infected sublingually with *C. albicans* strain 101. 21 days post-infection, mice were injected intraperitoneally with an anti-CD4 depleting antibody or an isotype control for two consecutive days. 7 days later, the frequency of CD3+CD8+ T cells among CD45+ cells was analyzed in blood (A), cervical lymph nodes (cLN, B) and tongue (C, left panel). In the tongue, absolute numbers of CD8+ T cells were also assessed (C, right panel). Data are the mean + SEM of 7 mice per group pooled from two independent experiments. Statistics were calculated using t-test, p*< 0.05, p***< 0.001.

Figure S6

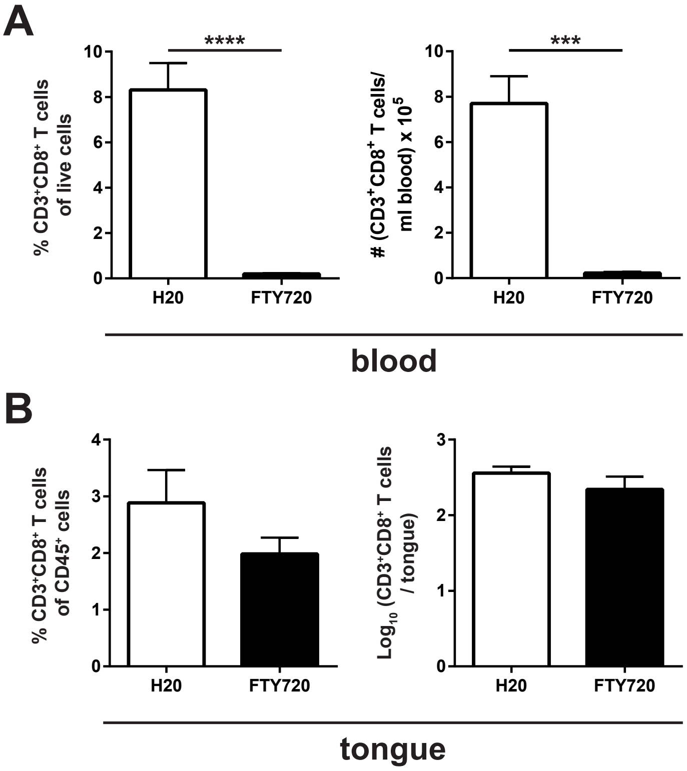

Figure S6 (related to Figure 5). Tongue CD8+ T cells in the tongue of C. albicans-colonized mice are not affected by FTY720. WT mice were infected sublingually with *C. albicans* strain 101. 21 days post-infection, FTY720 was administered in the drinking water for 3 weeks and CD3+CD8+ T cells in the blood and in the tongue were analyzed by flow cytometry. Frequencies (left) and absolute numbers (right) of CD3+CD8+ T cells in the blood (A) and in the tongue (B). Data are the mean + SEM of 10 mice per group pooled from two independent experiments. Statistics were calculated using t-test, p****<0.0001.

Figure S7

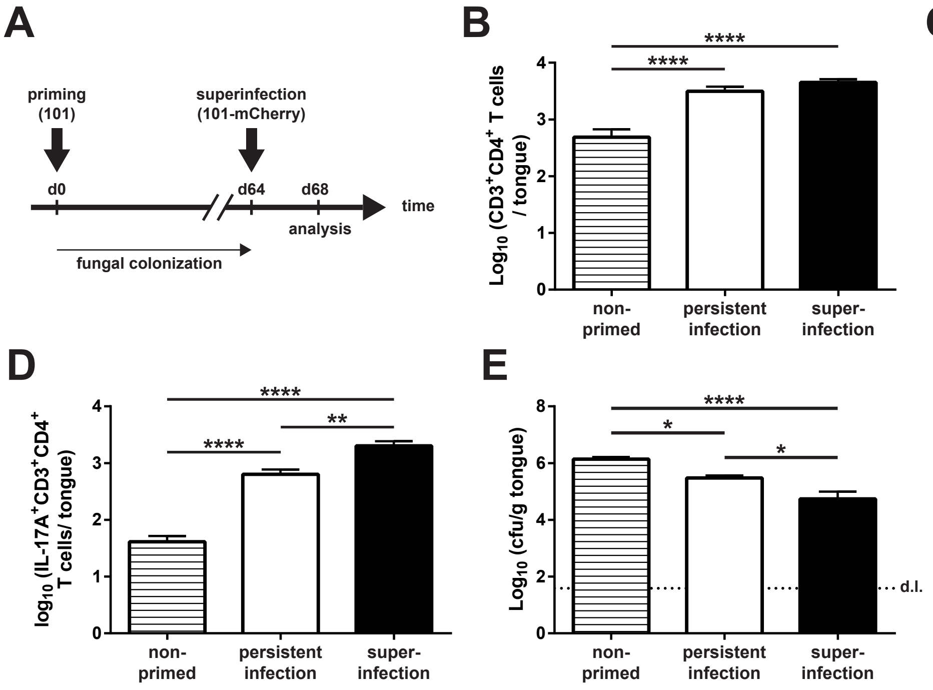

Figure S7 (related to Figure 6). TRM cells compensate fluctuations in the *C. albicans* colonization load. WT mice were infected sublingually with *C. abicans* strain 101 and super-infected with strain 101-mCherry on day 60 post-infection (superinfection) or not (persistent infection), as indicated. For the secondary infection, we used a mCherry-expressing variant of strain 101 to distinguish the fungus introduced during the primary and the secondary infection. As a control, a group of mice was included that did not receive a primary infection with strain 101 (non-primed). Tongue CD4+ T cells and fungal burden were analyzed on day 4 after (super)infection with strain 101-mCherry. (A) Schematic outline of the experiment. (B-C) Quantification of overall CD3+CD4+ T cells and CD103hiCD3+CD4+ T cells in the tongue. (D) Quantification of IL-17 production by CD3+CD4+ tongue T cells that were re-stimulated with PMA and ionomycin for 4 hours in the presence of Brefeldin A. (E) Tongue cfu. The dotted line represents the detection limit (d.l.). Each bar represents the mean + SEM of 7-13 mice per group pooled from 2-3 independent experiments. Statistics were calculated using one-way ANOVA, $p^{*} < 0.05$, $p^{**} < 0.01$, $p^{****} < 0.0001$.

Figure S8

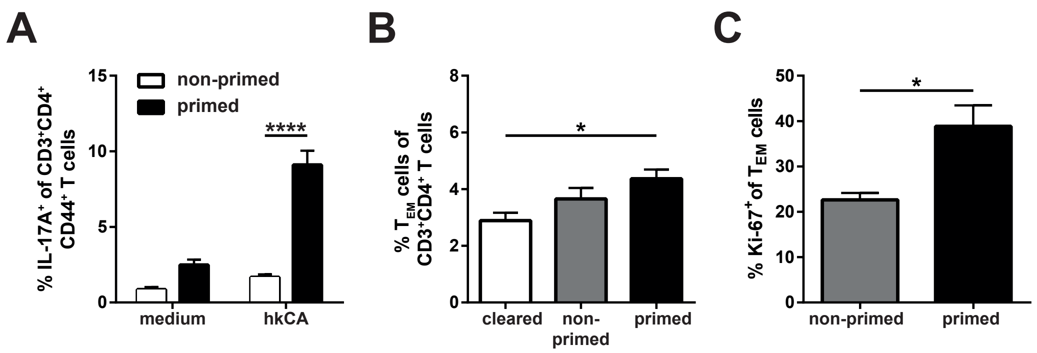

Figure S8 (related to Figure 7). TEM cells expand in the cervical lymph node after cleared infection and secondary fungal exposure. WT mice were infected sublingually with *C. albicans* strain 101 and treated with fluconazole for 2 weeks starting from day 8 post-infection onwards. Mice were re-infected on day 60 after the primary infection with strain 101-mCherry (primed). As a control, a group of naïve mice was included that did not receive a primary infection with strain 101 (non-primed). (A) To check for the efficiency of T cell memory induction 4 days after re-infection, cervical lymph node T cells were re-stimulated with *C. albicans*-pulsed DC1940 cells for 5 hours in the presence of Brefeldin A. IL-17A production was analyzed by intracellular cytokine staining and flow cytometry. Plots show the frequency of IL-17A-producing CD3+CD4+ T cells. Each bar represents the mean + SEM of 8 mice per group pooled from two independent experiments. (B, C) Plots depict the frequency of CD127+CD44+CD62L- TEM cells among CD3+CD4+ T cells (B). and the frequency of Ki-67+ TEM cells (C) in the cervical lymph nodes. Each bar represents the mean + SEM of 6-12 mice per group pooled from 2-3 independent experiments. Statistics were calculated using t-test (C), one-way ANOVA (B), or two-way ANOVA (A), p*< 0.05, p<*** 0.001.

Supplementary Table

REAGENT or RESOURCE	SOURCE	IDENTIFIER
**Antibodies (clones)**		
FITC anti-mTCRVα2 (B20.1)	Biolegend	Cat#127806, RRID: AB_ 1134188
FITC anti-mCD44 (IM7)	Biolegend	Cat#103022, RRID: AB_493685
FITC anti-mCD4 (RMA4.4)	Biolegend	Cat#116003, RRID: AB_313688
FITC anti-mCD11a (M17/4)	Biolegend	Cat#101106, RRID: AB_312779
FITC anti-mCD4 (RMA4.5)	Biolegend	Cat#100510, RRID: AB_312713
PE anti-mCD45.2 (104)	Biolegend	Cat#109808, RRID: AB_313445
PE anti-mIL-22 (Poly5164)	Biolegend	Cat#516404, RRID: AB_2124255
PE anti-mCD90.1 (H1S51)	eBioscience	Cat#12-0900-83, RRID: AB_465774
PE/Dazzle 594 anti-mCD103 (2E7)	Biolegend	Cat#121429, RRID: AB_2566492
PE/Cy5 anti-mCD3e (145-2C11)	Biolegend	Cat#100310, RRID: AB_312675
Pe/Cy7 anti-mIL-17A (TC11-18H10.1)	Biolegend	Cat#506921, RRID: AB_2125011
Pe/Cy7 anti-mCD69 (H1.2F3)	Biolegend	Cat#104512, RRID: AB_493564
PB anti-mCD4 (RM4-5)	Biolegend	Cat#100531, RRID: AB_493374
PB anti-mCD49a (Ha31/8)	BD Biosciences	Cat#740046, RRID: AB_2739815
BV421 anti-mIFN-γ (XMG1.2)	Biolegend	Cat#505830, RRID: AB_2563105
BV421 anti-mCD127 (A7R34)	Biolegend	Cat#135023, RRID: AB_10897948
BV570 anti-mCD90.2 (30-H12)	Biolegend	Cat#105329, RRID: AB_10917055
BV70 anti-mCD44 (IM7)	Biolegend	Cat#103037, RRID: AB_10900641
BV570 anti-mCD8α (53-6.7)	Biolegend	Cat#100739, RRID: AB_10897645
BV605 anti-mCD90.1 (OX7)	Biolegend	Cat#202537, RRID: AB_2562644
BV605 anti-mCD4 (RM4.4)	Biolegend	Cat#116027, RRID: AB_2800581
BV605 anti-mCD4 (RM4.5)	Biolegend	Cat#100548, RRID: AB_2563054
BV605 anti-mCD62L (MEL-14)	Biolegend	Cat#104438, RRID: AB_2563058
APC anti-mCD62L (MEL-14)	Biolegend	Cat#104412, RRID: AB_313099
APC anti-mTCRVα2 (B20.1)	Biolegend	Cat#127809, RRID: AB_1089251
APC anti-mCD45.2 (104)	Biolegend	Cat#109814, RRID: AB_389211
APC anti-mIFN-γ (XMG1.2)	Biolegend	Cat#505810, RRID: AB_315404
Alexa700 anti-mKi-67 (16A8)	Biolegend	Cat#652419, RRID: AB_2564284
Alexa700 anti-mCD90.1 (Ox-7)	Biolegend	Cat#202528, RRID: AB_1626241
Alexa700 anti-mCD45.2 (104)	Biolegend	Cat#109822, RRID: AB_493731
**Fungal Strains**		
C. albicans	[81]	SC5314
C. albicans	[45]	101
C. albicans	this report	mCherry-101
**Biological Samples**		
Fetal Calve Serum	Bioconcept	Cat# 2-01F10-I
**Chemicals, Peptides, and Recombinant Proteins**		
Phosphate Buffered Salt Solutions (PBS, 1x)	Amimed/Bioconcept	Cat# 3-05F39
RPMI 1640	Life Technologies	Cat# 21875034
2-Mercaptoethanol	Life Technologies	Cat# 31350-010
EDTA (0.5M, pH 8)	Life Technologies	Cat# AM9260G
Brefeldin A	Sigma-Aldrich	Cat# B6542-25MG
DNAse I	Sigma-Aldrich	Cat# DN25-100MG
Collagenase I	Life Technologies	Cat# 17100017
LIVE/DEAD Near IR Dead cell Stain Kit	Thermo Fisher	Cat# L10119
BD Cytofix/Cytoperm Reagent	BD Bioscience	Cat# 554714
eBioscience™ Foxp3 / Transcription Factor Staining Buffer Set	Thermo Fisher	Cat# 00-5523-00
BD Calibrite Beads	BD Bioscience	Cat# 349502
Paraformaldehyde	Sigma-Aldrich	Cat# P6148-500G
L-Glutamine	Thermo Fisher	Cat# X0550-100
Penicillin/Streptomycin	Amimed/Bioconcept	Cat# 4-01F00-H
Phorbol 12-myristate 13-acetate (PMA)	Sigma-Aldrich	Cat# P8139
Ionomycin	Sigma-Aldrich	Cat# I0634-1MG
Nonidet P40	Axonlab	Cat# A1694,0250
Ketasol	Graeub AG	Lot# 6680416
Rompun (2%)	Bayer	Lot# KP0BFHR
Fluconazole	Sigma-Aldrich	Cat# PHR1160-1G
Fingolimod (FTY720)	Selleckchem	Cat# S5002
FastStart Universal SYBR Green Master (Rox)	Roche	Cat# 4913914001
**Primer**		
Actb	[34]	fwd 5´-CCCTGAAGTACCCCATTGAAC-3´<br>rev 5´-CTTTTCACGGTTGGCCTTAG-3´
Itgae (CD103)	[88]	fwd 5´-CCTGTGCAGCATGTAAAAGAATG-3´<br>rev 5´-CCAGGATCGGCAGTTCAGATAC-3´
Klf2	[57]	fwd 5´-ACCAACTGCGGCAAGACCTA-3´<br>rev 5´-CATCCTTCCCAGTTGCAATGA-3´
S1pr1	[57]	fwd 5´-GTGTAGACCCAGAGTCCTGCG-3´<br>rev 5´-AGCTTTTCCTTGGCTGGAGAG-3´

**Cell Lines**		
DC^{1940} cells	Hans Acha-Orbea, University of Lausanne [85]	N/A
**Mouse strains**		
WT: C57BL/6JRj	Janvier Elevage	C57BL/6JRj
Rag1 $^{-/-}$ *:* B6.129S7-Rag1^{tm1Mom}/J	Swiss Immunological Mouse Repository (SwImMR) hosted by the University of Zurich, [76]	MGI:88666
TCRbd $^{-/-}$ *:* B6;129P2-TCRb^{tm1Mom} x TCRd^{tm1Mom}	Swiss Immunological Mouse Repository (SwImMR) hosted by the University of Zurich, [77,78]	MGI:1857256, MGI:1857257
Il17af $^{-/-}$: B6-Il17a/Il17f^{tm1Impr}	[79]	MGI:5438791
Il23r $^{GFP/GFP}$ mice	Burkhard Becher, University of Zurich, [80]	MGI:3844602
B6.SJL-*Ptprc* ^{a} *Pepc* ^{b} /BoyJ	Swiss Immunological Mouse Repository (SwImMR) hosted by the University of Zurich	N/A
**Software and Algorithms**		
GraphPad Prism V6	GraphPad	www.graphpad.com
NDP.view2.	Hamamatsu	U12388-01, www.hamamatsu.com
FlowJo V10	FlowJo LLC	www.flowjo.com
**Other**		
Gallios Flow Cytometer	Beckman Coulter	*www.beckmancoulter.com*
SP6800 Spectral Analyzer	Sony	*www.sony.com*
NanoZoomer 2.0-HT	Hamamatsu	www.hamamatsu.com
